# Link Between Personal History of Sexual Assault And Adhesion To Modern Myths About Sexual Aggression

**DOI:** 10.1192/j.eurpsy.2026.11811

**Published:** 2026-07-31

**Authors:** D. Triki, F. Guermazi, M. Abdelkafi, N. Regaieg, I. Feki, J. Masmoudi, I. Baati

**Affiliations:** Psychiatry A, Hedi Chaker university hospital, Sfax, Tunisia

## Abstract

**Introduction:**

Sexual assault is a traumatic experience that can have profound and lasting psychological consequences. Myths surrounding such a topic, which often blame the victim, can exacerbate the trauma of assault and create barriers to disclosure and seeking help. Understanding whether lived experience provides a form of defense against these myths is vital for improving support systems and informing educational strategies,especially for healthcare workers who may encounter victims in their practice.

**Objectives:**

Investigating links between having a personal history of sexual assault and the level of adherence to (AMMSA), including patterns of disclosure.

**Methods:**

A cross-sectional study was performed with 153 interns and residents. Using an online questionnaire, data were collected ( the participants’ history of sexual assault, the type of assault experienced, and whether they had disclosed the assault). The validated French AMMSA scale was used to quantify myth adherence.

Statistical comparisons of AMMSA scores were made using the appropriate tests.

**Results:**

A significant proportion of participants (20.9%, n=32) reported a history of sexual assault. The most common type was unwanted sexual touching (30.8% of those assaulted).

Analysis showed that participants without a history of sexual assault had significantly higher AMMSA scores (104.16 ± 24.33) than those with a history (92.78 ±23.5), with a p-value of 0.02. This difference was particularly pronounced for the myth endorsement following a history of sexual touching (88.3 ± 21.36 for those with a history vs 104.11 ± 24.54 for those without, p=0.007).

Furthermore, the majority of victims (51.3%) had never spoken to anyone about their assault, primarily due to fear of the reactions of those around them (60%).

**Conclusions:**

Having a personal history of sexual assault is associated with significantly lower adherence to myths about sexual aggression. This finding underscores that lived experience is a key resource in combatting harmful stereotypes. However, the pervasive fear of disclosure indicates that these myths still exert a powerful silencing effect.

**Disclosure of Interest:**

None Declared

